# FlyExpress 7: An Integrated Discovery Platform To Study Coexpressed Genes Using *in Situ* Hybridization Images in *Drosophila*

**DOI:** 10.1534/g3.117.040345

**Published:** 2017-06-30

**Authors:** Sudhir Kumar, Charlotte Konikoff, Maxwell Sanderford, Li Liu, Stuart Newfeld, Jieping Ye, Rob J. Kulathinal

**Affiliations:** * Institute for Genomic and Evolutionary Medicine and; ** Department of Biology, Temple University, Philadelphia, Pennsylvania 19122; † Department of Biomedical Informatics and; ‡ School of Life Sciences, Arizona State University, Tempe, Arizona 85287; § Department of Computational Medicine and Bioinformatics, University of Michigan, Ann Arbor, Michigan 48109; and † Center for Excellence in Genome Medicine and Research, King Abdulaziz University, Jeddah, Saudi Arabia

**Keywords:** dotplot, embryonic expression patterns, gene regulation, image analysis, intergenic regions

## Abstract

Gene expression patterns assayed across development can offer key clues about a gene’s function and regulatory role. *Drosophila melanogaster* is ideal for such investigations as multiple individual and high-throughput efforts have captured the spatiotemporal patterns of thousands of embryonic expressed genes in the form of *in situ* images. FlyExpress (www.flyexpress.net), a knowledgebase based on a massive and unique digital library of standardized images and a simple search engine to find coexpressed genes, was created to facilitate the analytical and visual mining of these patterns. Here, we introduce the next generation of FlyExpress resources to facilitate the integrative analysis of sequence data and spatiotemporal patterns of expression from images. FlyExpress 7 now includes over 100,000 standardized *in situ* images and implements a more efficient, user-defined search algorithm to identify coexpressed genes via Genomewide Expression Maps (GEMs). Shared motifs found in the upstream 5′ regions of any pair of coexpressed genes can be visualized in an interactive dotplot. Additional webtools and link-outs to assist in the downstream validation of candidate motifs are also provided. Together, FlyExpress 7 represents our largest effort yet to accelerate discovery via the development and dispersal of new webtools that allow researchers to perform data-driven analyses of coexpression (image) and genomic (sequence) data.

Gene regulation is a primary determinant of cellular differentiation, morphogenesis, and organismal adaptability. Its actions can be visualized by tracking the location and timing of functional products derived from genes and genomic elements. Studies on gene regulation in model organisms such as the fruit fly, *Drosophila melanogaster*, have established the successive dependence of gene expression of later developmental stages on earlier events the utilization of the same functional products in different ways at different times during development, and extensive coexpression patterns between genes (Stathopoulos and Levine, 2002a; Carroll *et al.*, 2005; Levine and Davidson, 2005; Matthews *et al.*, 2005; Weiszmann *et al.*, 2009; Frise *et al.*, 2010).

Fruit fly development is well-suited to study developmental gene networks and potential regulatory interactions. The volume of expression pattern data in the developing *D. melanogaster* embryo has increased drastically over the last 15 yr owing to individual and high-throughput efforts capturing the expression time-course of thousands of genes in the form of *in situ* images (Tomancak *et al.*, 2002, 2007; Lécuyer *et al.*, 2007; Fowlkes *et al.*, 2008; Frise *et al.*, 2010; Kumar *et al.* 2011). In addition, scores of studies have yielded experimentally verified functional and regulatory information for many developmental genes (Brody, 1999; Halfon *et al.*, 2008; Murali *et al.*, 2011; St Pierre *et al.*, 2014). Collectively, these data provide an unprecedented opportunity to identify coregulated genes and decode their underlying *cis*-regulatory logic by comparing spatial expression profiles, whose similarities provide an initial clue about potential joint roles in developmental processes (Kumar *et al.*, 2002; Lécuyer *et al.*, 2007; Weiszmann *et al.*, 2009; Frise *et al.*, 2010). In addition, the 2D *in situ* images themselves have proven to be a valuable resource for developing machine learning techniques to identify the developmental stages of embryonic images, generate initial text annotation terms describing expression patterns, and elucidate groups of coexpressed genes (Sun *et al.*, 2013; Jug *et al.*, 2014; Yuan *et al.*, 2014).

The FlyExpress knowledgebase (www.flyexpress.net) was developed to facilitate large-scale, data-driven (image-content) analyses of embryonic *in situ* expression patterns, and to accelerate the discovery of functional, genetic, and regulatory motifs that underlie interactions among genes and genetic elements. The basic FlyExpress platform now contains a unique digital library of standardized *in situ* images [> 100,000 images in > 4600 genes from the Berkeley *Drosophila* Genome Project (BDGP), Fly-FISH, and the literature] and a search engine to discover gene coexpression (Kumar *et al.*, 2011; Konikoff *et al.*, 2012). In developing the FlyExpress database, the existing data were enriched by standardizing each image so that they can be digitally aligned to each other (Kumar *et al.*, 2011), as well as annotating images to contain information on developmental stage range and anatomical orientation (*e.g.*, lateral, dorsal, and ventral). These enhancements allow researchers to easily visualize a temporal sequence of gene expression (*i.e.*, time-course embryos) for each gene, providing the necessary prerequisite for image comparisons, discovering coexpressed genes, and building informative global views of stage- and view-specific expression patterns. FlyExpress is integrated with, and complements, widely-used fly resources primarily containing textual information such as FlyBase (St Pierre *et al.*, 2014), the BDGP (Tomancak *et al.*, 2002, 2007), the Interactive Fly (Brody, 1999), REDfly (Halfon *et al.*, 2008), and FlyMine (Lyne *et al.*, 2007).

In this paper, we develop a new generation of analytical webtools in FlyExpress 7 that begins to integrate granular spatiotemporal expression patterns with genomic sequence data (Figure 1). FlyExpress 7 enhancements include: (i) a 30% increase in standardized *in situ* images, from primary literature found in PubMed; (ii) a new GEM-based algorithm that quickly and precisely identifies coexpressed genes defined by users at the temporal-cellular level; (iii) development and implementation of a unique “painting” tool permitting users to initiate a precisely controlled search of pixels across FlyExpress’ large standardized database of expression patterns; (iv) an interactive viewer of overrepresented shared DNA motifs that compares IGRs (intergenic regions) across user-defined pairs of genes; (v) link-outs to assist in the downstream validation of candidate motifs, including FlyBase, FlyMine, STRING, and Tomtom; (vi) an *in situ* image carousel allowing users to quickly compare hybridization images across developmental stages via an interactive animation feature for each gene (Animate); and (vii) a downloadable CSV gene list, providing users with summary results that can be immediately transferred to other databases. Together, these webtools enable researchers to better explore regulatory space and will ultimately lead to new hypotheses about developmental networks and their regulation.

**Figure 1:**
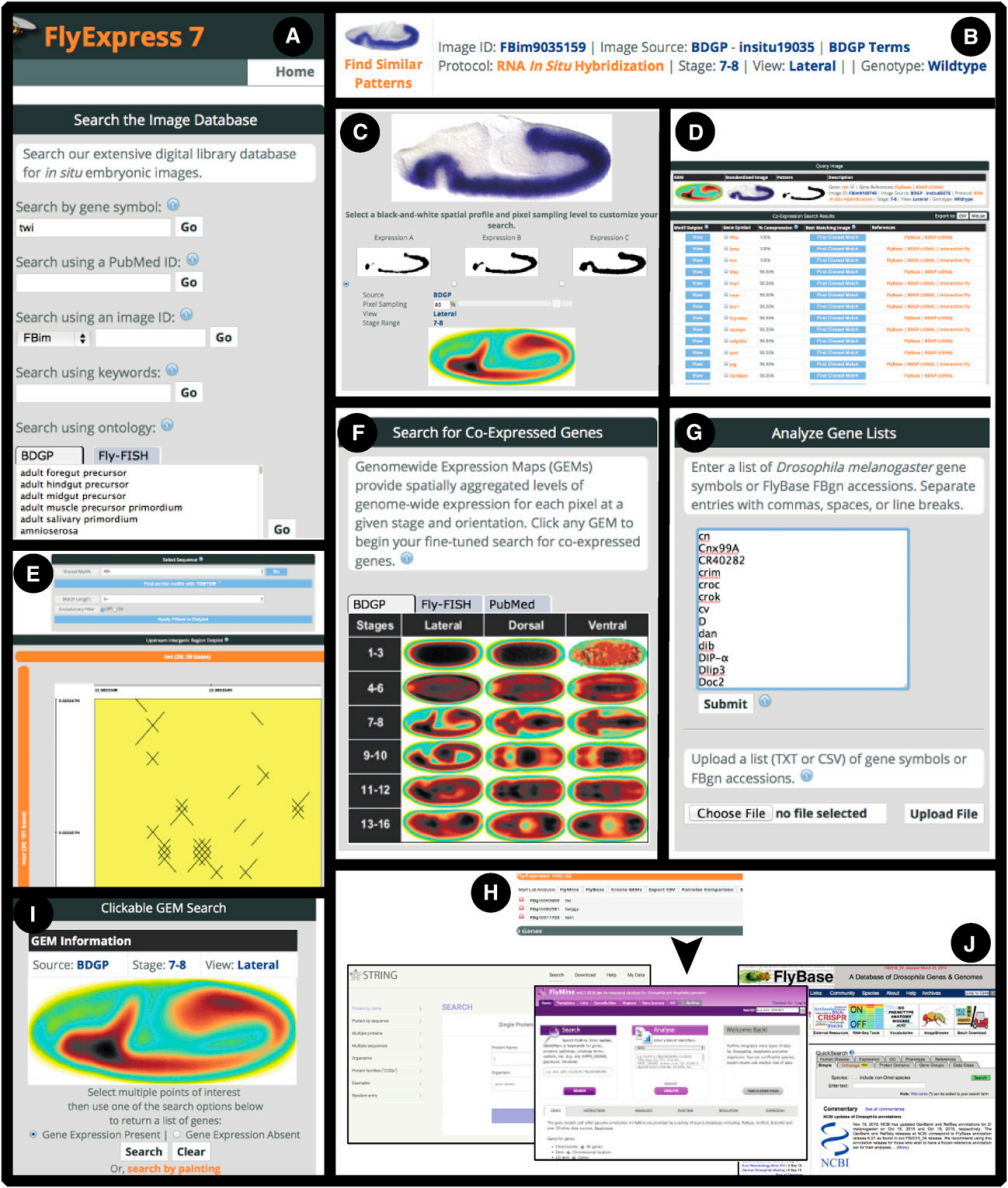
Integration of new discovery tools in FlyExpress 7 online platform for regulatory discovery. (A) Searching the FlyExpress digital image library reveals standardized *in situ* images for a gene or genomic element of interest. Along with gene names, queries can be also initiated by PubMed article ID, image ID, keywords, or anatomy ontology. (B) Each image in the digital library is annotated by data source, stage range, and anatomical orientation (*e.g.*, lateral, dorsal, or ventral). (C) Selecting an image along with an associated level of resolution initiates the search for coexpressed genes. (D) Results are displayed as a ranked list. Genes exhibiting the greatest coexpression are displayed first. Links to other *Drosophila* databases are provided for each gene. (E) Upstream IGRs of the query and result genes can be explored via an interactive shared motif viewer equipped with dotplot and genome browser. (F) GEMs provide another entry point to search for coexpressed genes by allowing global expression in the developing embryo to be rapidly visualized and searched for a given stage. (G) Clicking a pixel(s) of interest on the GEM returns a list of genes and genomic elements expressed at that precise location and stage. (H) A list of multiple genes can also be manually entered or uploaded. FlyExpress 7 automatically searches the digital library and adds them to the Hitlist. The Hitlist facilitates further analysis of coexpressed genes. (I) Custom GEMs can be manually created by selecting pixels across the embryo. Genes and genomic elements matching the user's selected expression pattern will be returned as a Hitlist. (J) The Hitlist content can also be exported to a number of popular resources for further analysis, including FlyBase (St Pierre *et al.* 2014), FlyMine (Lyne *et al.* 2007), and STRING (Franceschini *et al.* 2013). BDGP, Berkeley *Drosophila* Genome Project; GEMs, Genomewide Expression Maps; ID, identifier; IGR, intergenic region.

## Materials and Methods

### Curation of new images

The FlyExpress digital library currently holds 133,252 standardized images depicting *in situ* expression patterns of > 4500 embryo-expressed *D. melanogaster* genes. The majority (∼75%) of images come from the high-throughput BDGP and Fly-FISH (Lécuyer *et al.*, 2007) initiatives. New FlyExpress 7 images primarily originate from peer-reviewed PubMed papers (∼33,000 images from > 2800 publications published prior to 2007), and contain spatiotemporal patterns of a variety of genotypes. Black-and-white spatial profiles (representing gene expression in the developing embryo) were created for each image. The black-and-white spatial profiles translate into Binary Feature Vectors representing the composite spatial expression of each gene or genomic element (Gurunathan *et al.*, 2004). Images were annotated by genotype, image source, Campos-Ortega stage (Campos-Ortega and Hartenstein, 1985), and anatomical orientation. Incorporation of these new images has significantly expanded the size of the FlyExpress digital library and permits researchers to mine a more genotypically diverse collection of images.

### GEM-based searching

GEM-based searching in FlyExpress 7 replaces previous FlyExpress-implemented pairwise searches (Kumar *et al.*, 2011) used to discover coexpressed genes and genomic elements. Unlike traditional pairwise image searching, which necessitates precomputing an enormous pairwise matrix containing hundreds of millions of similarity values and requires a large-scale database indexing system (Kumar *et al.*, 2002; Konikoff *et al.*, 2012), this new GEM-based method is less computationally intensive and directly identifies coexpression via a predefined GEM. GEMs represent global expression in the developing embryo, and are unique for each collection of images belonging to the same data source, stage range, and anatomical orientation (Figure 1F). GEM-based searches are especially scalable to the large increase of available *in situ* embryo image data expected over the next few years.

GEM-based searching works by rapidly matching search pixels within standardized *in situ* images to discover genes exhibiting spatial patterns of coexpression at a given developmental time. Search pixels represent key points of embryonic expression, and allow researchers to quickly find specified regions of coexpression either by (1) manually pointing at and clicking on pixel(s) of interest or (2) by painting or highlighting regions of interest directly onto a GEM (Figure 1I). Coexpressed genes are identified by taking the collection of pixels in a black-and-white spatial profile, mapping them onto the appropriate GEM, and querying for genes that share the highest numbers of coexpressed pixels with the query. GEM-based coexpression between a query and result image is calculated by *n*/*t*, where *n* is the subset of search pixels exhibiting expression in both query and result images, and *t* is the total number of search pixels. By initiating the search through standardized GEM aggregates, the identification of similar images significantly reduces total computational effort, with coexpressed genes almost immediately returned to the user.

### Dotplot of intergenic regions of a pair of coexpressed genes

An interactive shared motif viewer (dotplot) was developed using custom code written in PHP (an HTML-embedded server-side scripting language) that renders as an SVG (Scalable Vector Graphic) markup to increase interactivity using native JavaScript Application Program Interfaces. From a queried gene, users can choose a coexpressed gene to compare upstream gene regions for structural genomic signatures of similarity. The dotplot function first queries FlyMine (Lyne *et al.*, 2007) for upstream IGRs for each of the pair of genes and uses the returned coordinates to fetch the corresponding sequence from a genomic sequence preloaded from FlyBase (St Pierre *et al.*, 2014). An evolutionary filter for the level of base pair conservation can also be implemented on the dotplot. To apply the evolutionary filter, an Asynchronous JavaScript and XML (AJAX) call is made to the FlyExpress server, which finds conservation values in the FlyExpress database for each base pair in the upstream region and regenerates the dotplot SVG by overlaying a gray-scale corresponding to the conservation for the region: 0–0.25 white, > 0.25–0.5 light gray, > 0.50–0.75 dark gray, and > 0.75–1 black. The dotplot displays averaged PhastCons (PHylogenetic Analysis with Space-Time models; Siepel *et al.*, 2005) 15-way scores for contiguous stretches of both query gene and result gene upstream IGRs. PhastCons assigns each *D. melanogaster* base pair a score between 0 and 1.

### Data availability

All data are available on the flyexpress.net website and can be provided upon request (s.kumar@temple.edu).

## Results

FlyExpress 7 includes novel, user-friendly webtools to facilitate the integrative analysis of sequence and coexpression data (Table 1), thereby distinguishing FlyExpress from previous releases as well as other *Drosophila* resources simply offering embryonic expression data, *e.g.*, FlyBase (St Pierre *et al.*, 2014), BDGP (Tomancak *et al.*, 2002, 2007), and Fly-FISH (Lécuyer *et al.*, 2007).

**Table 1:** Novel features and improvements in FlyExpress 7

	FlyExpress 6	FlyExpress 7
Digital library images	BDGP: 57,083	BDGP: 57,083
	Fly-FISH: 42,065	Fly-FISH: 42,065
	PubMed: 5,977	PubMed: 34,074
Coexpression searching	Pairwise	GEM-based
*In situ* image carousel	Absent	Present
Shared motif viewer	Absent	Present
Motif visualization	Absent	Interactive dotplot viewer
Hitlist functionality	Export to FlyBase and FlyMine	Export to FlyBase and FlyMine
	Downloadable gene list (CSV)	Downloadable gene list (CSV)
	Create custom GEMs	Create custom GEMs
		One-click export to STRING (Franceschini *et al.* 2013)
		One-click export to Tomtom (Gupta *et al.* 2007)

In addition to expanding the digital library, a variety of biologist-friendly tools integrating sequence analysis with temporal-spatial patterns of gene expression are available for use in FlyExpress 7. BDGP, Berkeley *Drosophila* Genome Project; GEMs, Genomewide Expression Maps.

### New GEM-based searching provides similar but faster results to pairwise searching

**Figure 2:**
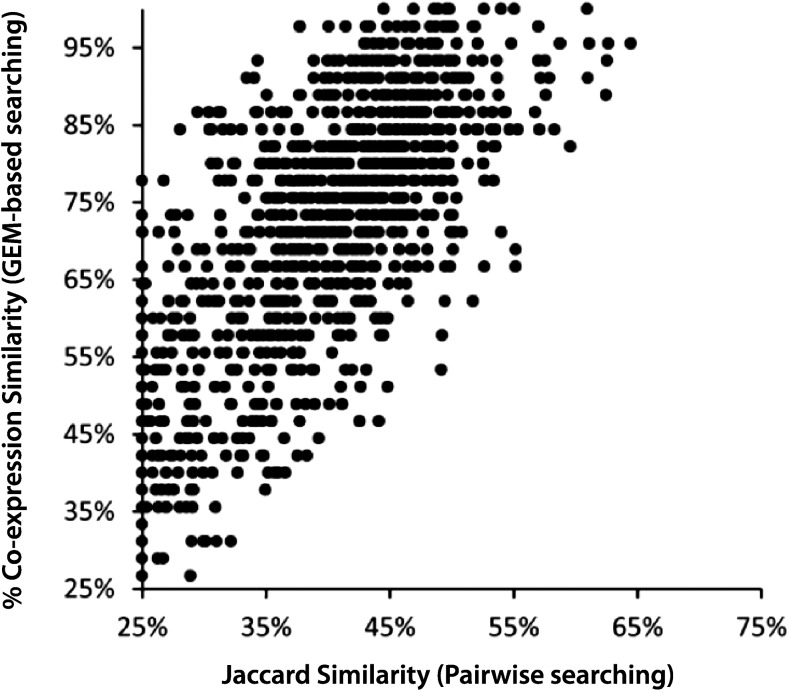
Pairwise *vs.* genomewide expression map (GEM)-based coexpression similarities for a query gene, *twi*, against all other genes. Both pairwise and GEM-based searches were performed to identify genes coexpressed with *twi* (FBim9035159_c, 40% pixel coverage; image shown in Figure 1C). For each coexpressed gene image matched against the *twi* image, GEM-based coexpression similarity is plotted against the Jaccard pairwise similarity. See Figure S1 for further analyses.

To determine how GEM-based searching compares to pairwise searching [see Kumar *et al.*, (2011)] in rapidly identifying coexpressed genes, we performed both searches using the same *twist* (*twi*) gene query image (Figure 2, FBim9035159_c, 40% pixel coverage). Pairwise and GEM-based searches both use black-and-white expression profiles, which permit the direct comparison of coexpression search results. Black pixels indicate expression presence and white pixels indicate expression absence. Pairwise similarity between two expression patterns is calculated by dividing the intersection of black pixels by the union of black pixels (Konikoff *et al.*, 2012). In GEM-based searching, selecting an expression profile generates a set of unique search pixels that represent key points of gene expression at a particular developmental stage. Coexpressed genes are found by taking the collection of black pixels in a spatial profile, mapping them onto the appropriate GEM (*i.e.*, same data source, stage range, and orientation), and identifying genes that share the highest numbers of coexpressed pixels.

A comparison of both search methods reveals that pairwise results return a subset of GEM-based results (Figure 2). For example, in the *twi* example at 40% pixel coverage, pairwise searching yields 980 genes exhibiting at least 25% similarity in coexpression. Of these, 103 have > 50% pixel similarity. All 980 genes were identified using the GEM-based searching method. GEM-based searching returns 1123 results coexpressed at ≥ 25% similarity of search pixels. Of these, 902 show ≥ 50% coexpression similarity. Interestingly, when repeated at twice the pixel coverage for *twi* (80% coexpression threshold), only 25 additional genes with ≥ 50% coexpression similarity appear among the GEM-based results (Supplemental Material, Figure S1, A and B). Overall, these results demonstrate a significantly greater return in similarly coexpressed genes using our new GEM search algorithms, and in a much faster time. Other gene queries performed with images from different data sources, stage ranges, and anatomical orientations produce similar results (Figure S1, C and D).

### Dotplot viewer helps identify conserved sequences and shared TFBS

**Figure 3:**
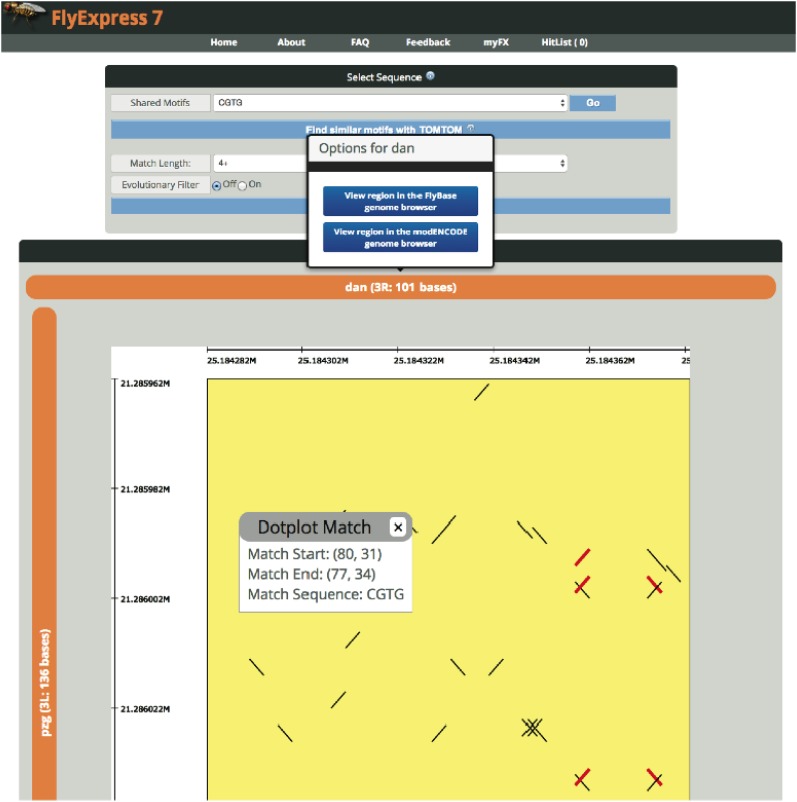
Shared motif viewer features and functionality. Biologists can visualize shared motifs from two upstream IGRs of a query gene and the selected coexpressed gene via a customizable dotplot. Shared motifs from the upstream intergenic regions of two genes, *distal anntena* (*dan*) and *putzig* (*pzg*), are displayed. Users can choose from precomputed shared motifs of a minimum k-mer length. An evolutionary filter can be applied to only include conserved sequence. Conservation is determined by averaged PhastCons scores (Siepel *et al.* 2005), with darker pixels indicating greater conservation (see *Materials and Methods* for details). Selecting a shared motif and clicking “Go” will highlight all matches in the dotplot viewer in red. The length of the IGR and genomic coordinates are indicated in the gene axis label. Clicking the gene name provides links to a choice of two genome browsers, FlyBase and modENCODE. Clicking a matched span (*i.e.*, contiguous line) in the dotplot will display a “Dotplot Match” context menu displaying start and end coordinates for the match as well as the matching sequence. IGR, intergenic region; PhastCons, PHylogenetic Analysis with Space-Time models.

From the query gene coexpression search results page, users can choose to view shared motifs between the query gene and a coexpressed gene of interest. Specifically, upstream IGR sequences belonging to both the query gene and a user-selected coexpressed gene, are compared via an interactive shared motif viewer (Figure 3). Users simply click “View” next to their coexpressed gene of interest and FlyExpress will generate a pairwise sequence dotplot based on each gene’s 5′ intergenic region.

The dotplot displays upstream IGR alignments for the query gene and chosen coexpressed gene. The default base pair filter setting displays contiguous stretches of 8-mers, but the length is customizable (Figure 3). Dotplot axis labels can be clicked to view upstream IGR sequences in FlyBase or modENCODE (Celniker *et al.*, 2009) genome browsers. Matches with at least the selected minimum length are displayed as contiguous lines whose sequences are listed in the “Shared Motifs” dropdown in the “Select Sequence” box at the top of the page (Figure 3). Selecting a shared motif and clicking “Go” will highlight all matches in the dotplot in red. In addition, clicking a matched span (*i.e.*, contiguous line) in the dotplot will bring up a “Dotplot Match” context menu displaying start and end coordinates for the match as well as the matching sequence (Figure 3). The full SVG representation of the dotplot can be downloaded by clicking “Download Dotplot” at the bottom of the page.

Base pair conservation can also be visualized on the dotplots via an evolutionary filter. Higher scores indicate greater conservation. Selecting a shared motif and clicking “Find similar motifs with TOMTOM” will submit the motif sequence to the Tomtom website (Gupta *et al*,. 2007) for additional analysis. Together with the evolutionary filters, the dotplot tool provides users with new candidate regions that may promote the precise control of spatial and temporal patterns of gene expression.

## Discussion

Embryogenesis is a complex and dynamic process that depends on the precise spatial and temporal regulation of gene expression across specific cell types. Understanding the regulatory elements and gene networks that underlie this heterogeneity in gene expression has been a major challenge in elucidating the genetic mechanisms involved in development and in understanding its underlying genomic and transcriptomic architecture. To help break this barrier, the FlyExpress knowledgebase was developed to assist researchers in rapidly mining available *in situ* images that capture the embryonic expression for thousands of *Drosophila* genes and genomic elements. Previous studies have used the FlyExpress knowledgebase to address such diverse problems from identifying target genes (*e.g.*, Deignan *et al.*, 2016) and generating genome-wide expression maps (*e.g* ., Ferrero *et al.*, 2014) to understanding patterns of paralog divergence (Konikoff *et al.*, 2012). With > 100,000 standardized *D. melanogaster* embryo images from > 4600 genes curated and collected across various platforms, including an almost sixfold increase in the amount of images extracted from the literature, we build new tools to expand the functionality of FlyExpress 7 beyond pure image-to-image analyses to containing tools that integrate sequence data with spatiotemporal patterns of expression.

Here, we briefly showcase the features and functionality of these new webtools and demonstrate their usefulness in allowing researchers to simultaneously visualize embryonic expression patterns and integrate genomic sequence analytical tools. Major site enhancements include a faster GEM-based search algorithm to identify coexpressed genes, a dotplot viewer between intergenic regions of a pair of genes, and direct link-outs to resources aiding in the downstream validation of candidate motifs and gene lists. As a result, FlyExpress provides a unique platform for users to identify genes with common regulatory features from its extensive database of curated images, visualize shared *cis*-regulatory motifs between any two genes for further study, and facilitate the seamless migration of results from one web platform to another (*e.g.*, FlyBase and FlyMine). Overall, this next generation of FlyExpress represents a turning point to integrate the *Drosophila* community’s unprecedented image-based resources with the generative potential of sequence data. Overall, these new tools and site enhancements represent our latest efforts to grow and evolve in response to the ever-changing needs of the fly community.

## Supplementary Material

Supplemental material is available online at www.g3journal.org/lookup/suppl/doi:10.1534/g3.117.040345/-/DC1.
