# Supplementary material for: FlyExpress 7: An Integrated Discovery Platform To Study Coexpressed Genes Using in Situ Hybridization Images in Drosophila
Source: G3 (Bethesda). 2017 Jun 30;7(8):2791–7. doi: 10.1534/g3.117.040345 (PMC5555482; doi:10.1534/g3.117.040345)
Supplement: Supplementary file 1 [file 2791FigureS1.docx]

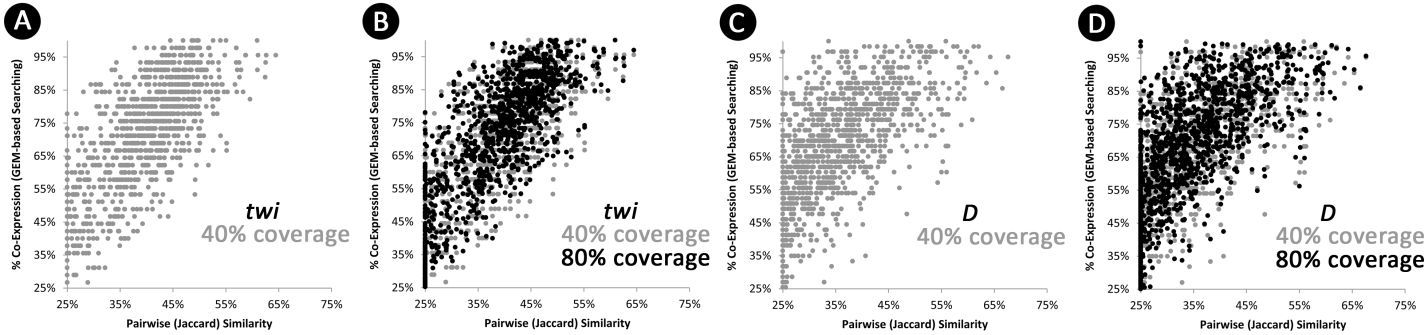


**Figure S1.** Pairwise (FlyExpress 6) vs. new GEM-based (FlyExpress 7) coexpression similarities for a queried gene against all other genes in FlyExpress. Pairwise and GEM-based searches were conducted with the same query image and spatial profile (i.e., pattern a, b, or c). For each search result, GEM-based co-expression similarities were plotted against pairwise (Jaccard) similarities. Values for the lateral stage 7-8 *twist* (*twi*) query (FBim9035159_c) at (A) 40% and (B) 80% pixel coverage are shown. Another gene query, *Dichaete* (*D*), at lateral stage 11-12 image (FBim9051495_c) for (C) 40% and (D) 80% pixel coverage yielded similar results.
